# Transcription factor AP2 enhances malignancy of non-small cell lung cancer through upregulation of USP22 gene expression

**DOI:** 10.1186/s12964-022-00946-9

**Published:** 2022-09-19

**Authors:** Ting Sun, Keqiang Zhang, Wendong Li, Yunze Liu, Rajendra P. Pangeni, Aimin Li, Leonidas Arvanitis, Dan J. Raz

**Affiliations:** 1 Division of Thoracic Surgery, City of Hope National Medical Center, Duarte, CA USA; 2 Laboratory of Surgery, The General Hospital of Ningxia Medical University, Yinchuan, China; 3 Present Address: Faculty of Health Science, University of Macau, Macau, China; 4 Faculty of Health Science, University of Macau, Macau, China; 5 Department of Pathology, City of Hope National Medical Center, Duarte, CA USA

**Keywords:** USP22, AP2, ATF3, NSCLC, Transcriptional regulation, Cell proliferation, Invasion, Malignancy

## Abstract

**Background:**

Ubiquitin-specific protease 22 (USP22), a putative cancer stem cell marker, is frequently upregulated in cancers, and USP22 overexpression is associated with aggressive growth, metastasis, and therapy resistance in various human cancers including lung cancer. However, *USP22* gene amplification seldom occurs, and the mechanism underlying USP22 upregulation in human cancers remains largely unknown.

**Methods:**

A luciferase reporter driven by a promoter region of *USP22* gene was selectively constructed to screen against a customized siRNA library targeting 89 selected transcription factors to identify potential transcription factors (TFs) that regulate USP22 expression in human non-small cell lung cancers (NSCLC). Association of identified TFs with USP22 and potential role of the TFs were validated and explored in NSCLC by biological assays and immunohistochemistry analysis.

**Results:**

Luciferase reporter assays revealed that SP1 and activating transcription factor 3 (ATF3) inhibit USP22 transcription, while transcription factor AP-2 Alpha/Beta (TFAP2A/2B) and c-Myc promote USP22 transcription. Binding site-directed mutagenesis and chromosome immunoprecipitation (ChIP) assays validated AP2α and AP2β are novel TFs of USP22. Furthermore, overexpression of AP2A and AP2B significantly upregulates USP22 expression, and its target: Cyclin D1, concurrently enhances the proliferation, migration, and invasion of NSCLC A549 and H1299 cells in a partially USP22-dependent manner. Moreover, AP2 protein level correlated with USP22 protein in human NSCLC tissues.

**Conclusion:**

Our findings indicate AP2α and AP2β are important transcription factors driving *USP22* gene expression to promote the progression of NSCLC, and further support USP22 as a potential biomarker and therapeutic target for lung cancer.

**Video Abstract**

**Supplementary Information:**

The online version contains supplementary material available at 10.1186/s12964-022-00946-9.

## Introduction

Ubiquitin-specific protease 22 (USP22) is a histone-modifying enzyme whose predominant function is the removal of the mono-ubiquitin moiety from lysine 120 of H2B (H2Bub1) [1, 2]. The *USP22* gene is located on short arm of the chromosome 17p11 and plays an important role in developmental and cancer biology through both histone ubiquitination dependent and independent pathways [3]. In cancer, USP22 was initially identified as one component of a “Death-By-Cancer**”** 11-gene signature associated with poor prognosis in a variety of cancers [4]. USP22 is frequently overexpressed in breast, colon, lung, and other cancers [1, 5–7]. USP22 is also considered a putative cancer stem cells (CSCs) marker in varous cancers [4, 8], while CSCs are known for therapy resistance and recurrence in cancers. Overexpressed USP22 is associated with chemotherapy resistance in hepatocellular, breast, and colorectal cancers [9, 10]. Mechanistically, USP22 stabilizes proteins or regulates activities of critical cancer drivers, including SIRT1, c-Myc, and CCND1, and inhibits many p53 host-protective functions [1, 11, 12]. USP22 affects DNA damage response (DDR), and USP22 is required for γH2AX deposition in response to DNA damage from irradiation [13]. USP22 knockdown markedly decreases cancer growth [1, 7, 11, 12]. Recently, we found that USP22 was frequently overexpressed in lung cancer and knockdown of USP22 suppressed CSC maintenance*, *in vivo angiogenesis, growth, and metastasis of KRAS mutant lung cancer, and significantly sensitized these cells to cisplatin and irradiation independent p53 status [14–16].

Regulation of USP22 expression is still poorly understood in cancer. The widely accepted finding of USP22 overexpression in cancer by immunohistochemistry (IHC) analysis is not consistent with the results of several additional genome-wide studies [14]. At the gene level, data from The Cancer Genome Atlas (TCGA) indicate that in many cancer types, the frequency of *USP22* somatic mutations is very low, and gene sequencing and mRNA expression data reveal that *USP22* gene is more frequently deleted or under-expressed than amplified or overexpressed in those same cancer types for which IHC studies found USP22 to be overexpressed [17–19], indicating transcriptional and or post transcriptional regulation of USP22 expression may be crucial to the level of USP22 protein in cancer. In terms of transcriptional regulation of USP22 expression, previous studies demonstrated that transcription factors SP1 [20] and cAMP-regulatory element-binding protein 1 (CREB1) [21] regulate USP22 gene expression. Recently, several micro RNAs (miRNAs) have been reported to inhibit the expression of USP22 in cancers [22–24]. At the protein level, knockdown of c-Myc was identified to decrease the protein level but not mRNA level of USP22 in acute myeloid leukemia stem cells [25].

Here, we investigated the underlying mechanism of USP22 expression in NSCLC using luciferase reporters driven by promoter regions of *USP22* gene with a panel of gene-specific siRNAs targeting these selected transcription factors (TFs) to identify potential TFs regulating USP22 expression in human NSCLC cells. We found that transcription factor 2 (TFAP2 also known as AP2), c-Myc, and nuclear transcription factor Y subunit alpha (NF-YA) promote the transcription of USP22 gene, while SP1 and activating transcription factor 3 (ATF3), a member of the ATF**/**CREB family of transcription factors, which may represent a potential tumor suppressor in lung cancer [26], inhibit the transcription of *USP22* gene in NSCLC cells. Given that the role of AP2 in USP22 expression has not been previously described, we further focused on regulation of USP22 by AP2. AP2 consists of five paralogs in mammals; AP2A to AP2E belonging to the AP2 transcription factor family [7]. In addition to their effects on development, AP2 family proteins also play an important role in cancer [27]. In this study, we for the first time showed that AP2 is an important transcription factor driving *USP22* gene expression; and revealed an association of AP2 with USP22 in lung cancer tissues, suggesting AP2 may promotes the progression of lung adenocarcinoma partially via enhancing USP22 expression.

## Materials and methods

### Patients selection and clinical data collection

This study was reviewed and approved by the Institutional Review Board (IRB) of City of Hope National Medical Center. A cohort of 240 NSCLC patients who underwent surgical resection with for curative intent between 2002 and 2014 without preoperative chemotherapy or radiation therapy were included, and patients’ clinical characteristics were reported previously [14]. Tissue microarrays were created using cancer and matched normal tissues.

### Cell lines

Human lung adenocarcinoma cell lines H1299 and A549 were purchased from ATCC. The H1299 cells were cultured in RPMI 1640 and A549 cells were cultured in DMEM supplemented with 10% FBS and 100U/ml penicillin and streptomycin. Both cells were authenticated by the Integrative Genomics Core by using short-tandem repeat polymorphism analysis before use. Cell lines were tested mycoplasma free using colorimetric mycoplasma detection assay with HEK-Blue-2 cells as described before [28]. *USP22* knockout (USP22−/−) cells were generated from H1299 and A549 cell lines by CRISPR/Cas 9 system as previously described [14].

### Genomic DNA extraction and Plasmid constructions

Genomic DNA was isolated from H1299 cells using DNA extraction Kit (QIAGEN). The promoter region from -5085 to + 55 of the *USP22* gene was cloned and constructed into PGL 4.14 vector by Kpn I and Xho I (NEB). Other deletion fragments were also cloned into PGL4.14 and sequences were acquired by PCR using the -5085/ + 55 plasmid as a template. Primers are shown in Additional file 1: Table S1. Mutation plasmid was constructed by using F6R0-PGL4.14 as a template, using mutagenesis kits from TAKARA following the manufacturer’s instructions. The forward primer is 5’-CGGGATTGGTATTGGCTTGCAGGCTCCCCTC-3’; the reverse primer is 5’-CAAATACCAATCCCGAGCTGCGCCTGCTG-3’. Total RNA was extracted from H1299 cells and reverse-transcribed using RT-Kit (QIAGEN). Whole length of TFAP2A and TFAP2Β were amplified by PCR and constructed to pcDNA3.1 vector, separately. Primers are shown in Additional file 1: Table S1.

### Transfection, Luciferase reporter assay and siRNA screening

H1299 and A549 cells were plated in plates 24 h before being transfected with *USP22* promoter-driven luciferase reporter plasmids and Renilla-luciferase expressing plasmid in a 10:1 ratio by using lipofectamine 3000. For siRNA screening, 3000 cells for each well were mixed with transfection master mix, and then seeded in 96 well plates. Forty-eight hours after transfection, cells were washed with PBS and lysed for 15 min in room temperature. Supernatant was then analyzed by TECAN plate reader for both Firefly and Renilla luciferase activity according to the manufacturer’s instruction of Dual-luciferase reporter system (Cat #: E1910, Promega). The list of siRNA library targeting for transcription factors was shown in Additional file 1: Table S2. For validation, additional siRNAs targeting TFAP2A, TFAP2B and c-Myc were purchased from Santa Cruz Biotechnology.

### Cell proliferation, migration and invasion assays

Cells were seeded in 6-well plates and transfected with siRNA or plasmids. Cell proliferation, migration and invasion assay were performed as described previously [28].

### Western blots and Immunohistochemistry analysis

Total cellular protein was extracted with SDS lysis buffer and heated in 95 °C for 7 min. Electrophoresis was performed and proteins were transferred onto PVDF membranes using Thermo Fisher i-Blot machine. Membrane was blocked by 5% milk in PBST for 1 h at room temperature and was incubated with primary antibody overnight. After washing out the primary antibody, the membrane was incubated with a secondary antibody for 1 h and sent for imaging. A primary antibody against β-actin (1:3000), c-Myc, AP2A, AP2B, and FLAG-HRP was purchased from Santa Cruz Biotechnology. Primary antibody against USP22 was purchased from Abcam. Antibody against cyclin D1 was purchased from Cell Signaling Technology.

### ChIP assay and quantitative reverse transcription PCR (qRT-PCR) assay

ChIP assays were performed using EZ-ChIP Kit (Millipore) according to manufacturer’s instructions. 1 × 10^7^ cells were cultured in 10-cm plate. Cells were fixed by adding formaldehyde to a final concentration of 1% for 10 min and glycine was added to neutralize untreated formaldehyde. Cells were washed, lysed and collected in 1.5 ml tube and sonicated under 4 °C to shear the cross-linked DNA to 200–1000 bp. The cross-linked protein-DNA was immunoprecipitated with magnetic beads, and a combination of anti-AP2α and AP2β antibodies (these two TFs bind to the same DNA sequence), and a normal IgG as a control for nonspecific binding at 4 °C overnight with rotation. The beads containing protein-DNA complex were pulled down by magnet holder. After several washing steps, the purified protein-DNA complexes were eluted. The cross-link of protein-DNA complexes were reversed by 5-h incubation and rotation at 65 °C with proteinase K. qPCR was performed using primers for the *USP22* promoter region. Primers used for qPCR are: forward primer: 5’- CGACACCCTCCGCGAACC -3’ and reverse primer: 5’- CGGAGGCCGGACAAAGAT -3’.

### Analyzing TFAP2 mRNA expression in Lung Adenocarcinoma (LUAD) using publicly available TCGA gene expression data

The Cancer Genome Atlas (TCGA) lung cancer Illumina HiSeq normalized RNA sequencing (LUAD, n = 513) data were downloaded from the University of California Santa Cruz Xena Browser (xenabrowser.net/datapages/) for correlation expression analysis by running the ggscatterstats function of the R package ggstatsplot (https://github.com/IndrajeetPatil/ggstatsplot).

### Statistical analysis

All experiments were performed in duplicates or triplicates and repeated at least two times. All graphs were prepared with GraphPad Prism version 8.0.2 (GraphPad Software, Inc.). Statistical analysis was performed using Student’s t-test for group–group analysis and Spearman correlation was performed for correlation studies.

## Results

### Characteristics of *USP22* promoter

**Fig. 1:**
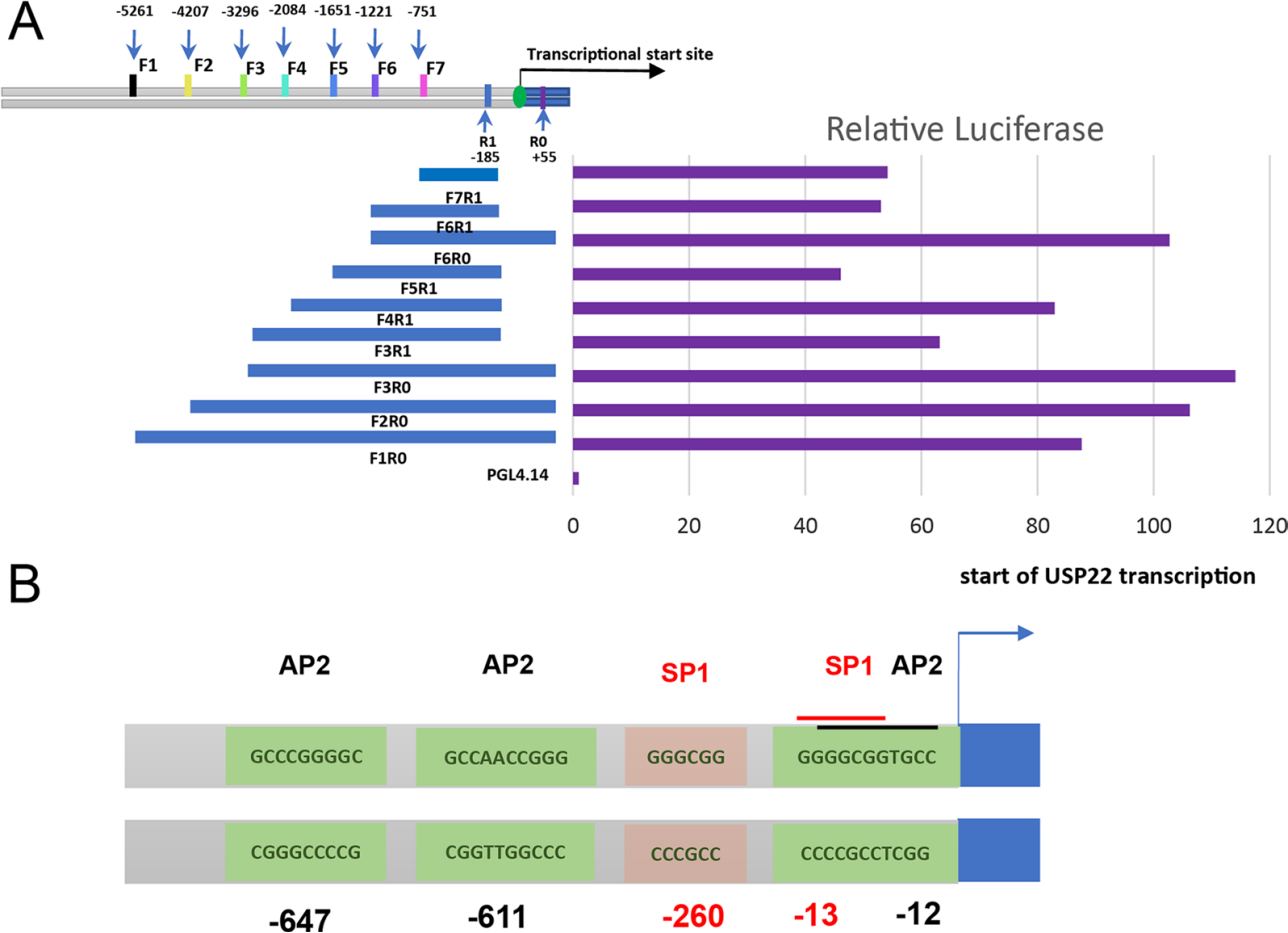
Characteristics of USP22 gene promoter. **A** Positions of USP22 promoter regions used for constructing luciferase reporters and transcriptional activity of these luciferase reporters in lung cancer H1299 cells. **B** Binding sites and sequences of transcription factors SP1 and AP2 in USP22 promoter region

To characterize TFs that regulate expression of *USP22* gene, we uploaded the sequenced −5261/+55 region in human *USP22* promoter to PROMO website [29] to predict the potential TFs binding to the *USP22* promoter, and about 136 TFs including the reported SP1 and CREB (Additional file 1: Fig. S1) were identified. Based on this analysis, multiple truncated fragments of the *USP22* gene promoter were amplified from genomic DNA of human lung cancer cell H1299 to construct various promoter region-driven luciferase reporter plasmids. These reporter plasmids were transfected into H1299 cells with CMV-promoter-driven renilla luciferase plasmid in a 10:1 ratio. Both firefly and the reference renilla luciferase were measured. As shown in Fig. 1A, the longest construct, p-5261/+55, which named as F1R0, showed a more than 80-fold increase in luciferase activity of PGL4.14-Basic constructs. The F2R0 (− 4207 to + 55), F3R0 (− 3296 to + 55) and F6R0 (− 1221 to + 55) also displayed higher luciferase activities of > 100 times of the basal, indicating that the region from -5261 to -3296 may inhibit the promoter activity of the *USP22* gene. F3R0 had drastically higher luciferase activity compared with F3R1, suggesting that − 185 to + 55 region was essential to transcriptional activity of *USP22* promoter, and the difference in the reporter activity between F6R0 and F6R1 also supported this finding. F4R1 showed higher activity than F3R1 and F5R1, indicating that the region from -3296 to -2084 may also bind suppressive TFs, while the region from -2084 to -1651 enhanced luciferase activity. F5R1, F6R1 and F7R1 luciferase activity was not significantly different, which means the fragment from − 1651 to − 751 was not essential for *USP22* promoter activity in NSCLC. The region R1-R0 (− 185/+ 55) was critical to *USP22* transcriptional activity. As shown in Fig. 1B, we found that there was only one binding site for AP2 that is located at − 12 bp/− 3 bp very close to the transcriptional start site and overlaps with SP1 binding site − 13 bp/− 8 bp.

### Identification of TFs regulating *USP22* transcription in lung cancer cells

**Fig. 2:**
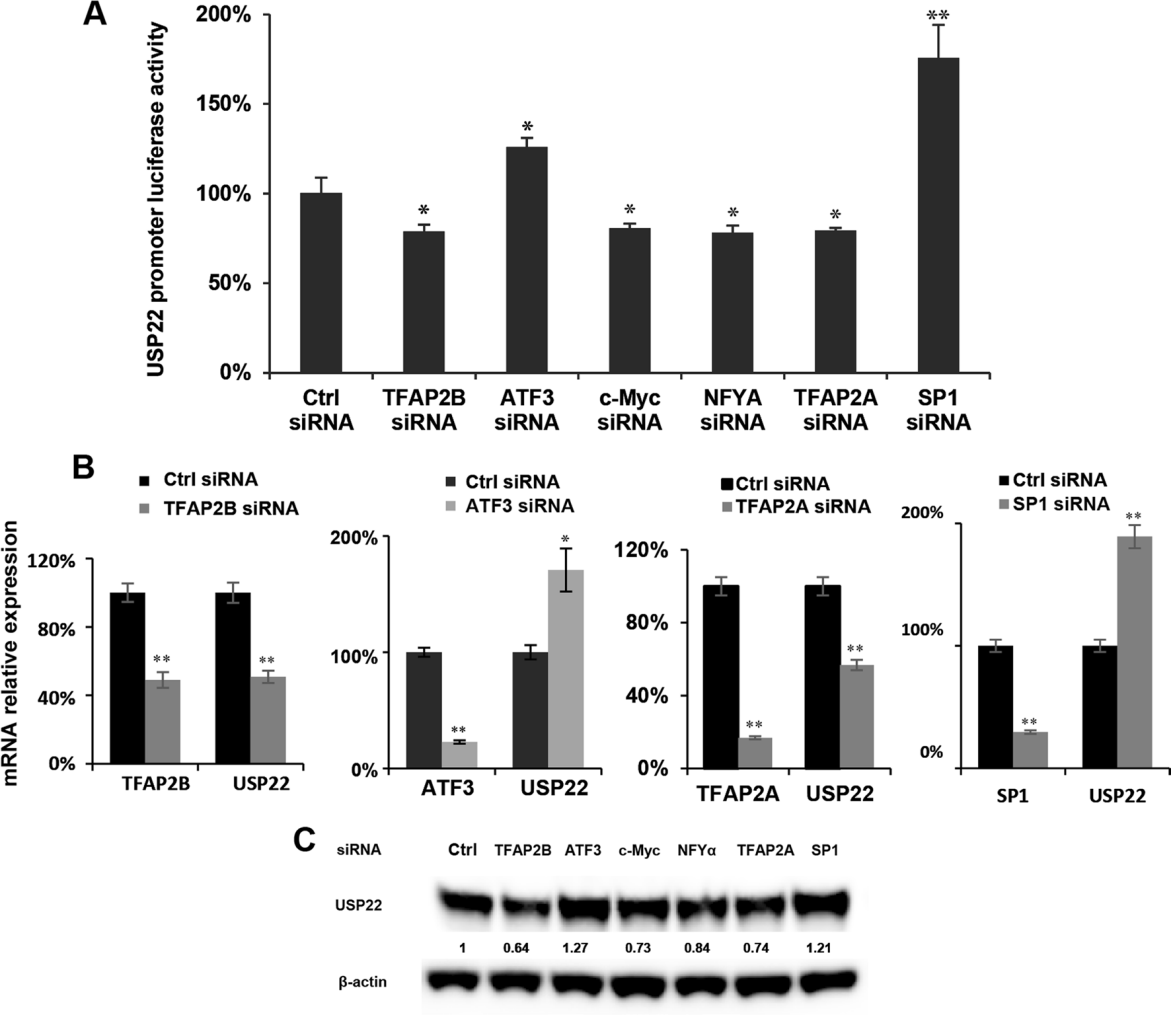
Identification of transcription factors that regulate USP22 gene expression in lung cancer cells. **A** Knockdown of transcription factors of TFAP2A/2B, c-Myc, NF-YA decreased the luciferase activity of USP22 promoter, while knockdown of transcription factors of SP1 and ATF3 increased the luciferase activity of USP22 promoter. **B** USP22 mRNA change by knockdown of TFAP2, ATF3 and SP1 in lung cancer cells; *P < 0.05, **P < 0.01, compared to the control siRNA. **C** Western blot analysis shows USP22 protein change upon knockdown of these transcription factors in lung cancer cells

Based on characterization of *USP22* promoter region; a customized siRNA library (Themo Fisher) including 89 validated siRNAs targeting known TFs and factors potentially binding to the region (Using virtual binding programs described previously) was generated (Additional file 1: Table S2). Each TF was knocked down using its gene-specific siRNA to examine its impact on the luciferase activity of F1R0 (− 5261/+ 55) promoter in H1299 lung cancer cells. Luciferase reporter assay identified that knockdown of *AP2A/2B*, c-Myc, NF-YA significantly suppressed *USP22* promoter-driven luciferase activity, while knockdown of SP1 and ATF3 dramatically enhanced *USP22* transcription (Fig. 2A). These findings were then further validated by qRT-PCR (Fig. 2B) and Western-blot (Fig. 2C) analysis, demonstrating that knockdown of TFAP2A, TFAP2Β, and c-Myc siRNA consistently decreased the mRNA and protein level of USP22, while knockdown of ATF3 and SP1 significantly upregulated *USP22* mRNA and protein levels (Fig. 2B, C). In addition, NF-YA was found to suppress USP22 luciferase activity but resulted in only a moderate decrease in mRNA and protein of USP22 (mRNA data not shown).

### C-Myc upregulates USP22 in lung cancer cells

**Fig. 3:**
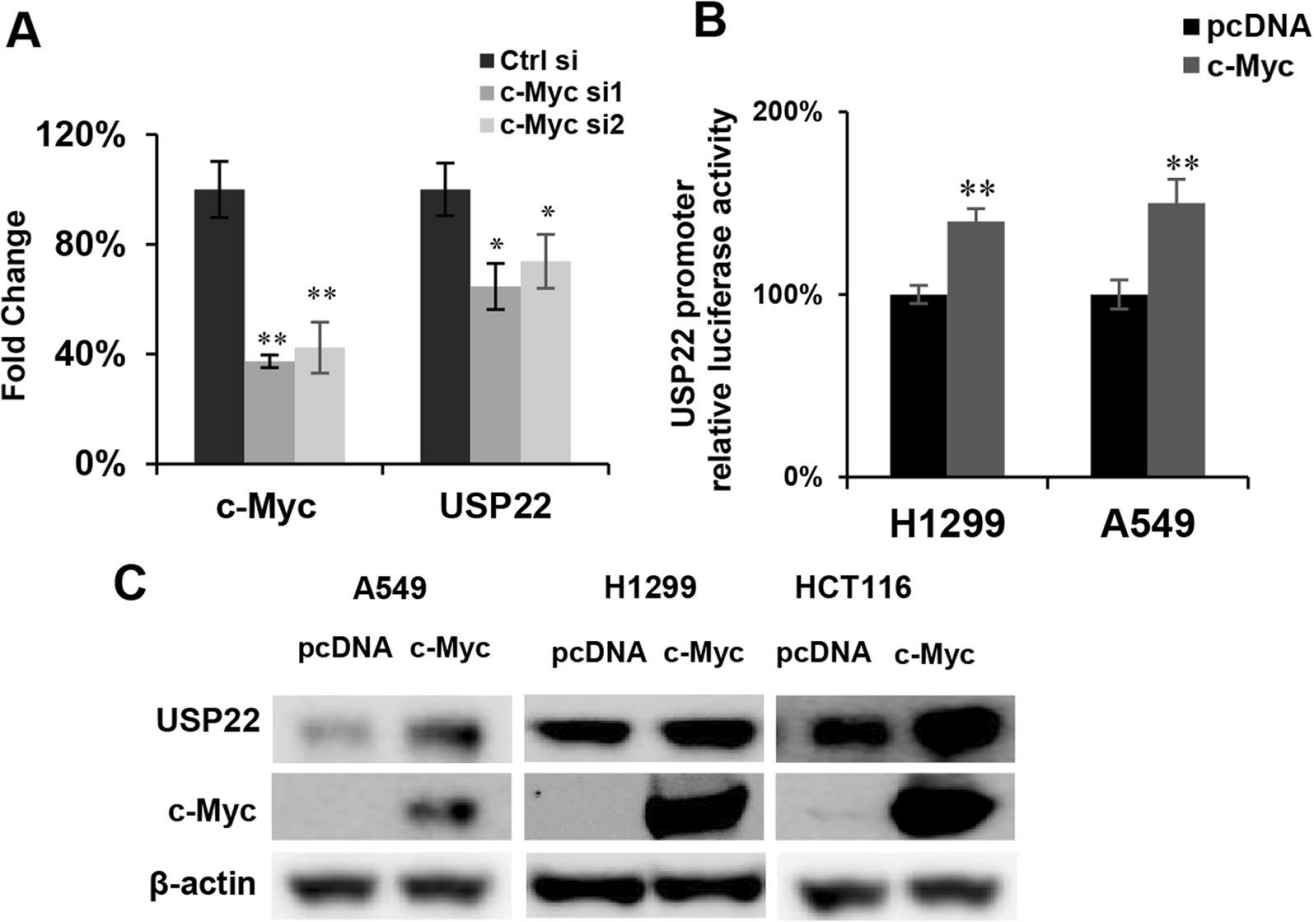
c-Myc upregulates USP22 expression in lung cancer cells. **A** knockdown of c-Myc by two individual gene-specific siRNA decreased USP22 mRNA; *P < 0.05, **P < 0.01 compared to the control siRNA. **B** Overexpression of c-Myc increased UPS22 promoter-driven luciferase activity of in both H1299 and A549 lung cancer cells; **P < 0.01 compared to the control pcDNA empty plasmid. **C** Overexpression of c-Myc upregulated USP22 protein in H1299 and A549 lung cancer cells and HCT116 colorectal cancer cells

Considering the critical role of c-Myc oncogene in cancer, we herein further investigated the impact of c-Myc on *USP22*-promoter-driven luciferase activity using an additional siRNA to knock down c-Myc in H1299 cancer cells. The analysis showed that two individual c-Myc specific siRNAs consistently decreased *USP22* mRNA expression (Fig. 3A). Furthermore, we found that c-Myc overexpression significantly upregulated both *USP22* promoter-driven luciferase activity (Fig. 3B) and USP22 protein levels in two lung cancer cells (A549, H1299) and a colorectal cancer cell line HCT116 (Fig. 3C). These data indicate c-Myc is also an important TF promoting *USP22* gene expression in lung cancer cells.

### Knockdown of *TFAP2A* and *TFAP2Β* downregulated USP22 expression and suppressed cell proliferation

**Fig. 4:**
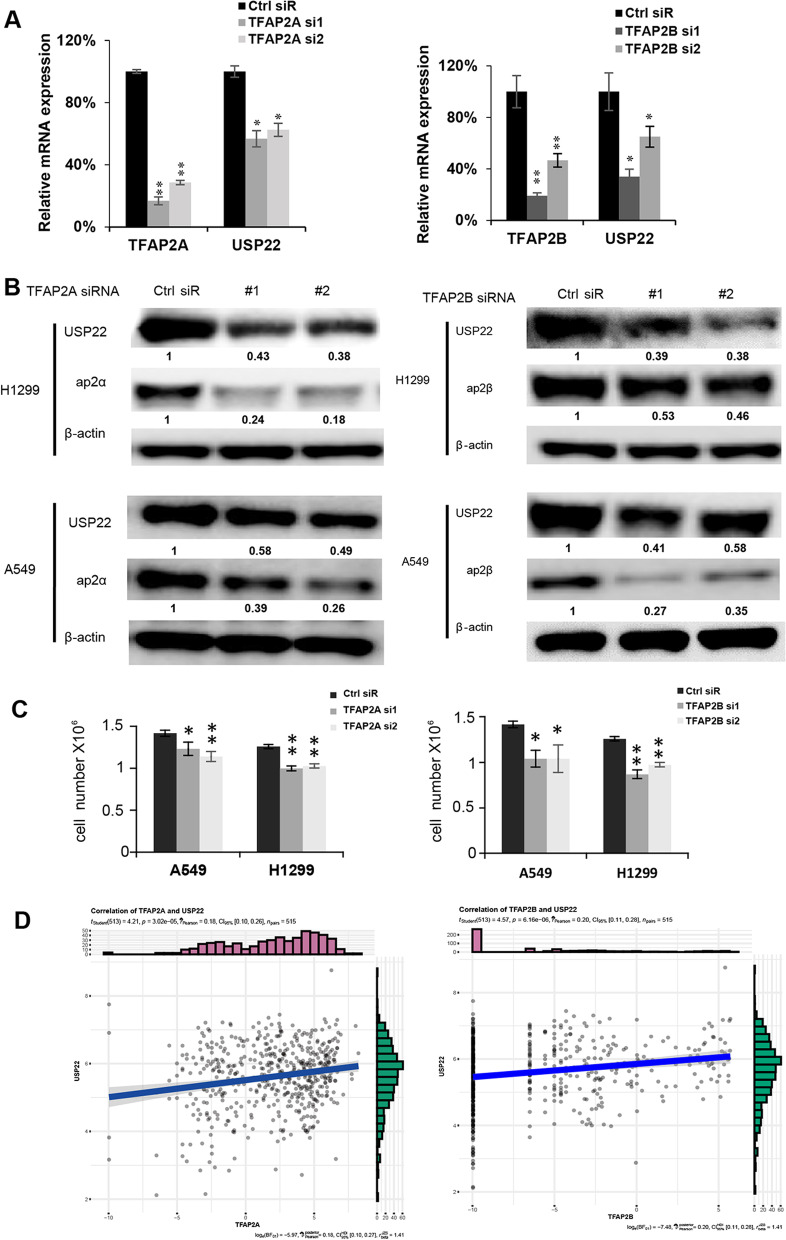
Knockdown of TFAP2 downregulated USP22, suppressed cell proliferation of NSCLC cells. Knockdown TFAP2A (left panel) and TFAP2B (right panel) by siRNA decreased **A** USP22 mRNA (*P < 0.05, **P < 0.01 compared to the control siRNA) and **B** USP22 protein in H1299 and A549 cells. **C** Knockdown of TFAP2A (left panel) and TFAP2B (right panel) decreased the proliferation of A549 and H1299 lung cancer cells; *P < 0.05, **P < 0.01 compared to the control siRNA. **D** The ggscatterstats diagram shows the positive association of the USP22 mRNA to TFAP2A mRNA (left panel) and TFAP2B mRNA (right panel) in human lung cancer tissues

To further confirm the role of AP2A and AP2Β in USP22 expression, additional siRNA for *TFAP2A* and *TFAP2Β* were synthesized and transfected to H1299 (upper panel) and A549 (lower panel) cells, and the results showed that two individual specific siRNAs targeting AP2A (left panel) and AP2Β (right panel) markedly decreased mRNA expression (Fig. 4A, data of A549 wasn’t shown) and protein levels (Fig. 4B) of USP22 in H1299 and A549 lung cancer cells. The impact of AP2 knockdown on cell proliferation was also measured, and the results indicated that knockdown of AP2A (left panel) and AP2B (right panel) resulted in a significant decrease of cell proliferation in H1299 and A549 cells at 72 h post-transfection of siRNA (Fig. 4C). We also analyzed mRNA expression in TCGA data and found there was a moderate correlation between USP22 mRNA expression with both AP2A (Fig. 4D, left panel) and AP2Β (Fig. 4D, right panel) expression in human lung cancer tissues, indicating AP2 may enhance USP22 transcription in lung cancer tissues.

### AP2 regulates USP22 activity by directly binding to promoter of *USP22*

**Fig. 5:**
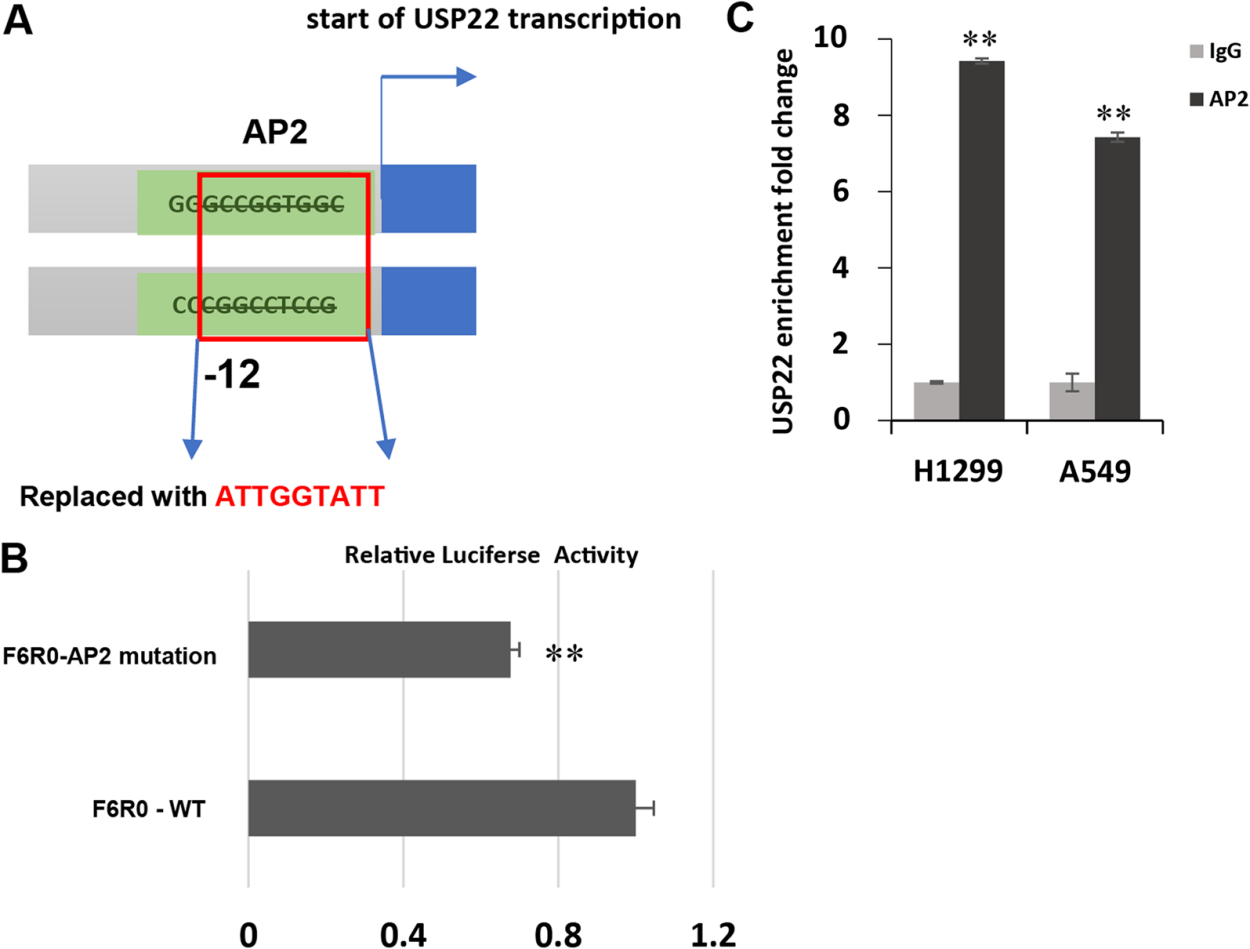
AP2 binds to USP22 promoter. **A** Diagram for mutated AP2 binding site (-12 to -4). **B** Mutation of AP2 binding site resulted in decreased luciferase reporter activity of USP22 promoter (**P < 0.01 compared to the wild-type USP22 promoter driven luciferase reporter). **C** ChIP assay shows compared to IgG, a mixture of anti-AP2α and AP2β antibody pulled down significantly more USP22 promoter DNA in H1299 and A549 lung cancer cells (**P < 0.01 compared to the control IgG)

As described above, we found that there was 1 binding site for AP2 located at − 12 bp/− 3 bp which is very close to the transcriptional start site and it also overlaps with the SP1 binding sequence. We hypothesized that AP2 TFs regulate *USP22* by directly binding to the promoter region of *USP22*. To test this hypothesis, we first constructed a luciferase reporter of *USP22* promoter harboring a mutated AP2 binding site from F6R0, which covered − 1221/+ 55 region by replacing the -12/-3 region “GCCGGTGGC” with “ATTGGTTTA” (Fig. 5A), then compared with the wild type promoter to find that luciferase activity of the mutated promoter reporter was drastically decreased (Fig. 5B). Furthermore, ChIP assay was performed to validate the binding of AP2 to the *USP22* promoter. A mixture of AP2α and AP2β antibodies was used to pull down the DNA sequence. As shown in Fig. 5C, AP2 antibodies successfully pulled down the promoter region of *USP22* in both H1299 cells and A549 cells. Compared with the nonspecific binding of IgG to the *USP22* promoter, there was a tenfold increase of the *USP22* promoter region DNA being pulled down by AP2 specific antibodies, indicating specific binding. In summary, the above data demonstrated that the AP2 protein regulates *USP22* gene expression in lung cancer cell line through transcriptional regulation.

### AP2A and AP2B involved in lung cancer cell malignancy partially through USP22

**Fig. 6:**
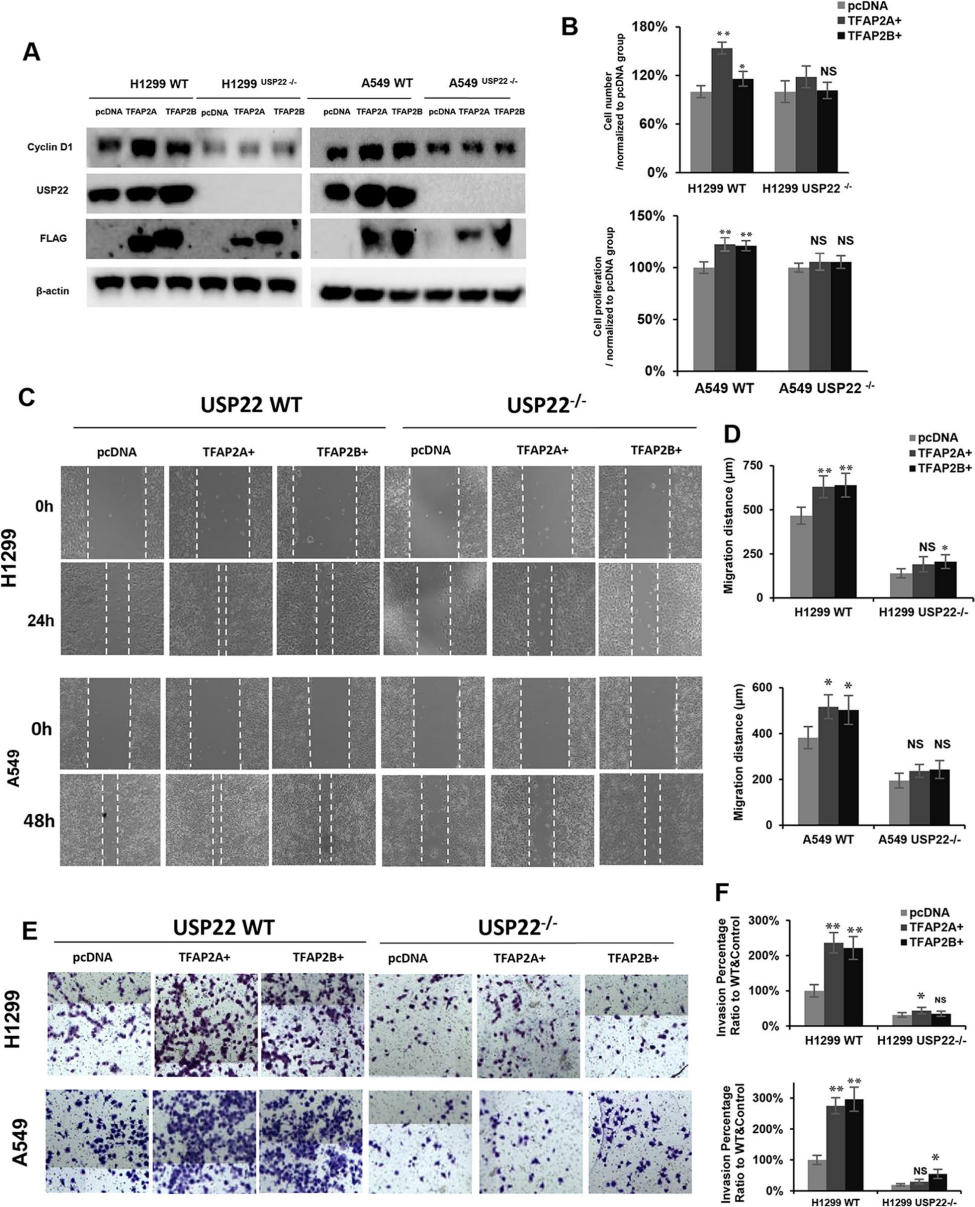
Overexpression of AP2 enhances malignancy of lung cancer cells. Overexpression of AP2A (left panel, shown by anti-Flag antibody) and AP2B (right panel, shown by anti-Flag antibody) increased **A** USP22 Cyclin D1 proteins and **B** Proliferation in the parental (USP22-WT) but not the USP22-knockout (-KO, −/−) H1299 and A549 cells. **C** Overexpression of AP2 increased the migration of USP22-WT but not USP22-KO H1299 and A549 cells. **D** Quantitative data of migration assays. **E** Overexpression of TFAP2 increased the invasion of USP22-WT but not USP22-KO H1299 and A549 cells. **F** Quantitative data of invasion assays; *P < 0.05, **P < 0.01 compared to the pcDNA empty plasmid

The biological significance of the AP2 protein was further explored in human lung cancer cells. As shown in Fig. 6A, overexpression of AP2 drastically upregulated USP22 protein as well as Cyclin D1 (a downstream target of USP22) in both H1299 (left panel) and A549 (right panel) cancer cells, while overexpression of AP2 did not upregulate Cyclin D1 in USP22 knock-out (USP22−/−) H1299 and A549 cancer cells, (which was previously generated and reported [14]). Notably, overexpression of AP2 also significantly enhanced the proliferation of USP22-WT H1299 (upper panel) and A549 (lower panel), but not in USP22-KO H1299 and A549 cancer cells (Fig. 6B). Interestingly, migration (Fig. 6C, D) and invasion (Fig. 6E, F) assays also demonstrated that overexpression of AP2 significantly enhanced cellular migration and invasion potential in the USP22-WT H1299 and A549 cancer cells, but had little effect on migration and invasion in the USP22-KO lung cancer cells. These data indicate that USP22 is a critical target of AP2 in lung cancer cells.

### Expression of AP2 is correlated with USP22 in lung cancer tumor tissues

**Fig. 7:**
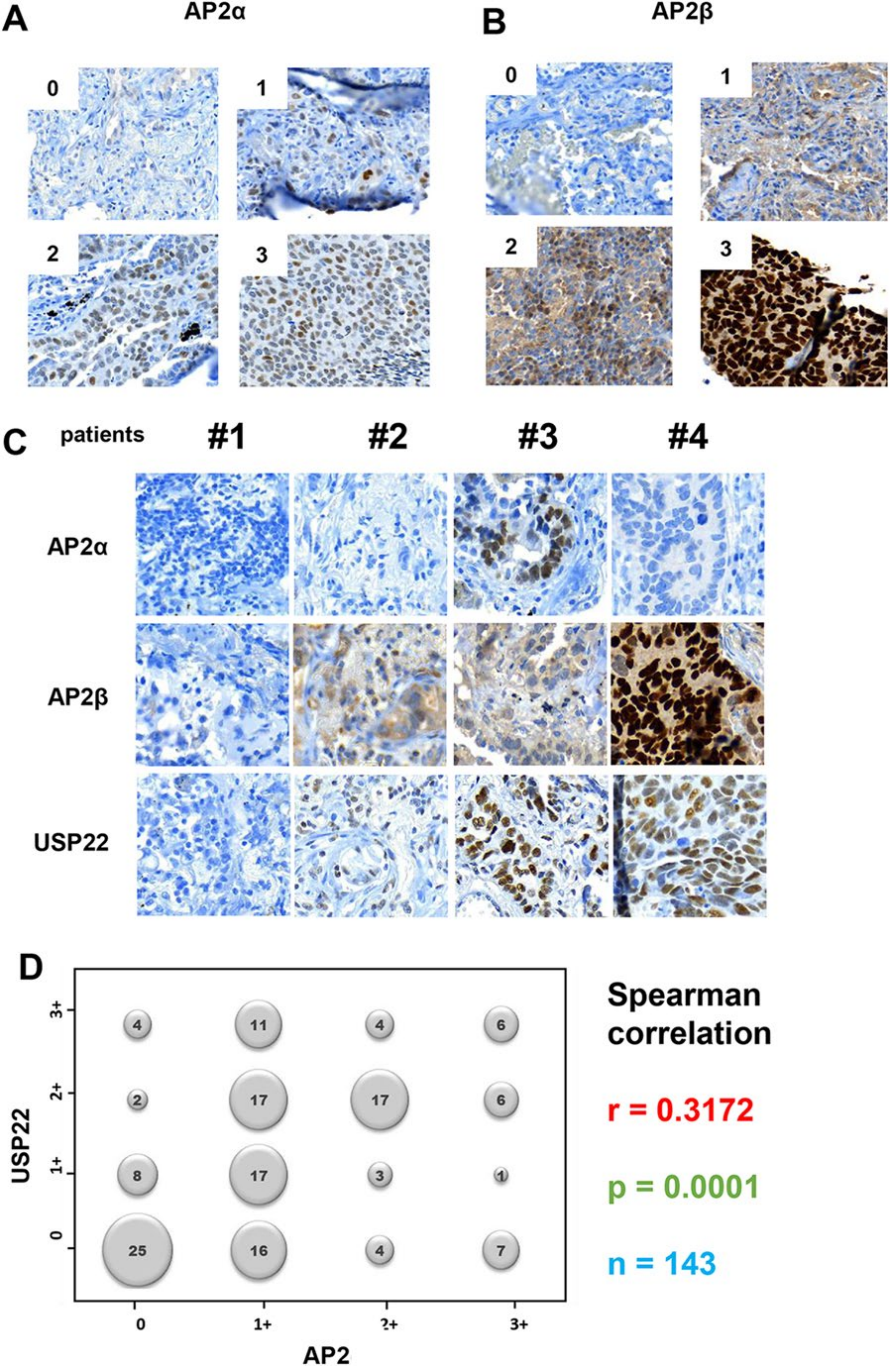
Expression of AP2 is correlated with USP22 in lung cancer tissues. Representative scored IHC staining of **A** AP2α, **B** AP2β. **C** Representative images of USP22, AP2α, and AP2β IHC staining in the same lung cancer tissues; 0: < 1%, 1 + : 5%, 2++: 5–25%, 3+++: > 25% positively stained cancer cells. **D** Spearman correlation analysis shows AP2 protein (a combination of AP2α and AP2β) was positively correlated to USP22 protein in 143 NSCLC tissues

We next investigated the expression patterns and levels of AP2α and AP2β in lung cancer patient tissues by IHC analysis. As shown in Fig. 7A, AP2α showed moderate nuclear staining in cancer tissues. Semi-quantitative analysis of IHC analysis showed that AP2α was undetectable (scored as 0) in 82.6% (128/155) of cancer tissues, while AP2α levels in 9.7% (15/155), 6.5% (10/155), and 1.3% (2/155) of 155 cancer tissues were scored as 1+, 2+, and 3+ respectively. While AP2β showed both cytoplasmic and nuclear staining in most lung cancer tissues Fig. 7B, AP2β was undetectable (scored as 0) in 30.9% (47/155), while AP2β levels in 46.1% (70/155), 15.8% (24/155), and 7.2% (11/155) of cancer tissues were scored as 1+, 2+, and 3+ respectively. Of note, both AP2α and AP2β proteins are rarely detected in the paired normal lung tissues (Additional file 1: Fig. S2). In Fig. 7C, co-expression pattern of AP2α, AP2β and USP22 was shown in 4 representative tissues, demonstrating that a positive of either AP2α or AP2β IHC staining was frequently associated with a higher level of USP22 expression in lung cancer tissues. When we analyzed data of combined AP2α and AP2β protein expression (either or both positive). Spearman correlation analysis demonstrated a moderate correlation between AP2 TFs and USP22 (r = 0.32, *p* < 0.001, Fig. 7D), suggesting that USP22 overexpression in lung cancer tissues is partially due to increased prevalence of the AP2 TF family.

## Discussion

In this study, we found that AP2, ATF3, c-Myc, NF-YA, and SP1 are critical to *USP22* mRNA expression in lung cancer cells. Moreover, immunohistochemical analysis shows that there is a positive correlation between AP2 TF and USP22 in lung cancer tissues, consistent with the oncogenic role of USP22 in lung cancer. We have further demonstrated that AP2 is associated with malignancy of lung cancer. Our findings, for the first time, demonstrate that AP2 is an important transcription factor driving *USP22* gene expression, promoting the progression of NSCLC in part by enhancing USP22 expression, which will further facilitate the understanding of the USP22 network and the targeted treatment of lung cancer.

In the study, by bioinformatics analysis, we found that within the region from -3296 to + 55 of *USP22* gene promoter, there are multiple putative binding sites for transcription factors SP1, ATF3, and Myb, c-Myc, and AP2, and confirmed these transcription factors regulated USP22 expression by a combination of *USP22*-promoter and luciferase reporter and specific sRNA knockdown assays. We herein focused on AP2 because it has not been previously described in the regulation of USP22. In terms of transcriptional regulation of USP22 expression, prior studies have demonstrated that transcription factors SP1 [20], CREB1 [21], and HIF1a [30] regulate USP22 transcription. It was shown that a promoter region from − 210 to + 52 contains the basal promoter activity of USP22 in colorectal cancer Hela cell [20]. Surprisingly, oncogene SP1 was found to negatively regulate USP22 expression. At the transcriptional level, Sp1, which binds to a site just ahead of the *USP22* transcription start site (− 13 to − 7), is an important negative regulator of *USP22* transcription. Similarly, in this study, we also found critical transcriptional inhibitors may be present in − 185 to + 55, and the data from the luciferase reporter assay also indicated that potential transcription factors binding to other sites outside of this region may promote USP22 transcription. We found that there are several binding sites of AP2 transcription factor in the *USP22* promoter region, and an AP2 binding site close to upstream of the *USP22* transcriptional start site (− 12 to − 3) that almost overlaps with the SP1 binding site, is critical to enhance *USP22* transcription. We found that mutation or deletion of these motifs, particularly those closest to the translational start site, resulted in a significant decrease in *USP22* promoter activity, suggesting that the AP2 promotes *USP22* gene transcription.

Consistent with the potential oncogenic role of USP22 in lung cancer, AP2 also appears to play a positive role in tumorigenesis. Therefore, loss of activity of AP2 can lead to the impairment of proliferation, differentiation and apoptotic processes, suggesting AP2 activity may play a role in development [22]. AP2 proteins have been identified to regulate the expression of many downstream genes, thereby participating in a variety of cell processes, particularly cell differentiation, survival, and proliferation as well as apoptosis within various developmental contexts [7]. TFAP2A and TFAP2C have been shown to participate in tumorigenesis by modulating the expression of many cancer-related genes, such as VEGF, P21, Rb, TP53, BCL2, c-KIT, MMP-2, E-cadherin, and c-Myc [27]. AP2 family proteins are often overexpressed in human cancer. Expression levels of TFAP2A and TFAP2C are higher in both adenocarcinoma and squamous cell lung cancer tissues than in normal lung tissues [31, 32], and AP2 overexpression is associated with tumorigenesis and aggressive biology in lung cancer [31, 32]. TFAP2A induced Keratin-16 overexpression, promotes tumorigenicity in lung adenocarcinoma and metastasis via epithelial-mesenchymal transition (EMT) [33]. Similarly, another study demonstrated that AP2α could potentially promote lung adenocarcinoma metastasis possibly by triggering EMT [34]. AP2 family proteins were also considered a prognostic factor for cancer including lung cancer. In nasopharyngeal carcinoma, APα was demonstrated to regulate the growth and survival through modulation of the HIF-1α-mediated VEGF/PEDF signaling pathway [35]. TFAP2A was related to an unfavorable overall survival in lung cancer and its upregulation was significantly related to the overall survival rate in patients that smoked or underwent non-chemotherapy and non-radiotherapy procedures [31]. AP2β overexpression has been found to promote tumor growth in both breast and thyroid cancer and predicted poor prognosis or tumor progression, respectively [36, 37]. Similarly, AP2α overexpression is recognized as a prognostic indicator of shorter patient survival in epithelial ovarian carcinomas [38]; while strong AP2β expression showed a positive correlation with the poor prognoses of patients with lung adenocarcinomas [39]. Consistent with the above findings, in the present study, we showed that both AP2α and AP2β proteins were overexpressed, and there was a positive correlation between USP22 and AP2 proteins in lung cancer tissues, and we also demonstrated that knockdown of TFAP2A and TFAP2B significantly suppressed the proliferation and invasion potential of lung cancer cells.

Besides AP2, we found that ATF3, a member of CREB protein family of transcription factors may suppress *USP22* gene expression, and it seems ATF3 may possess an opposite transcriptional role of the previously reported CREB1 in USP22 expression [21]. Interestingly, a previous study revealed that ATF3 acts as a metastatic suppressor and patients with high adenylate kinase-4 (AK4) and low ATF3 expression showed unfavorable outcomes compared with patients with low AK4 and high ATF3 expression [40]. In addition, ATF3 decreases nuclear factor kappa B subunit 2 (NFKB2) expression and reverses drug resistance in lung cancer cells [41]. These observations indicate that ATF3 may function as a tumor suppressor in lung cancer. NF-YA is also found to be involved in USP22 expression in lung cancer, while global overexpression of NF-YA is documented in lung adenocarcinoma and is associated with aggressive cancer behavior [42]. Therefore, the contribution and regulation of these two transcription factors to USP22 expression may also deserve further investigation.

At the posttranscriptional level, several studies revealed the regulation of USP22 protein by other signals or proteins. We previously showed that cisplatin upregulates USP22 protein in lung cancer stem cells [15], indicating that activation of DNA-damage-repair pathways may potentially promote USP22 protein through either translational or post translational mechanisms. In addition, we found that c-Myc, which can be stabilized through deubiquitination by USP22 [43], upregulates USP22 at both transcriptional and post transcriptional levels, suggesting existence of a positive feedback loop between USP22 and c-Myc in lung cancer. Meanwhile, a previous study revealed that c-Myc knockdown resulted in a decrease in USP22 protein levels, but does not change its mRNA level [25]. This inconsistency may be caused by different cancer cells used in each study and or experimental variations, since we found that the dynamic changes in *USP22* mRNA occurred quickly, but lasted only a short time after stimulation or modulation. In addition, APC/C was reported to promote degradation of USP22 during the cell cycle [44].

In summary, our results demonstrate that USP22 expression is promoted by AP2 and c-Myc and is suppressed by SP1 and ATF3 at the transcriptional level. AP2 is an important transcription factor driving USP22 expression, and it may enhance malignancy of NSCLC partially through upregulating *USP22* transcription. Given the important roles of both USP22 and AP2 in lung cancer carcinogenesis, our finding of the association between these two proteins further supports USP22 deubiquitinate as a potential biomarker and therapeutic target for lung cancer.

## Supplementary Information

**Additional file 1**. Supplementary figures and tables.

## Data Availability

Not applicable.

## Abbreviations

USP22: Ubiquitin-specific protease 22
NSCLC: Non-small cell lung cancers
ATF3: Activating transcription factor 3
TFAP2A/2B: Transcription factor AP-2 Alpha/Beta
H2Bub1: Mono-ubiquitin moiety from lysine 120 of H2B
CSC: Cancer stem cells
DDR: DNA damage response
IHC: Immunohistochemistry
TCGA: The Cancer Genome Atlas
CREB1: CAMP-regulatory element-binding protein 1
TFs: Transcription factors
NF-YA: Nuclear transcription factor Y subunit alpha
ChIP: Chromosome immunoprecipitation
LUAD: Lung adenocarcinoma
