# Supplementary material for: Transcription factor AP2 enhances malignancy of non-small cell lung cancer through upregulation of USP22 gene expression
Source: Cell Commun Signal. 2022 Sep 19;20:147. doi: 10.1186/s12964-022-00946-9 (PMC9484186; doi:10.1186/s12964-022-00946-9)
Supplement: Supplementary file 2 — Additional file 1. Supplementary figures and tables. [file 12964_2022_946_MOESM2_ESM.pdf]

FigureS1

A

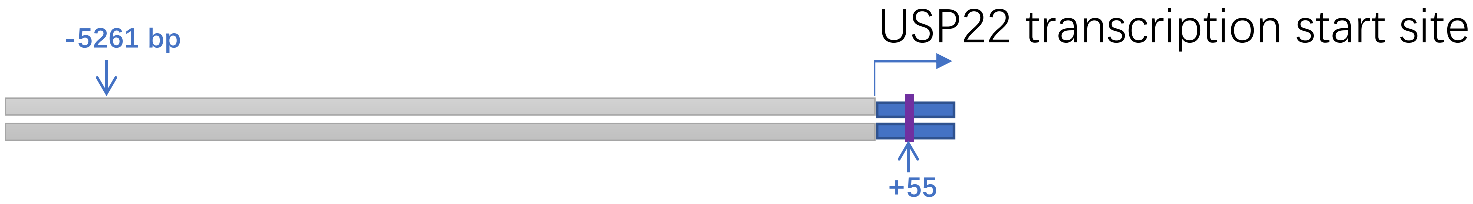

Transcriptional factors prediction

B

0	POU1F1a [T0069	1	C/EBPbeta [T00581]	2	C/EBPalpha [T001	3	NF-Y [T00150]	4	NF-1 [T00539]	5	GR-alpha [T00	6	TFII-I [T00	7	NF-AT2 [T01945]
8	STAT4 [T01577]	9	NF-AT1 [T01948]	10	NF-AT1 [T01944]	11	c-Ets-1 [T00112]	12	YY1 [T00915]	13	STAT1beta [T0	14	c-Jun [T00	15	c-Myb [T00137]
16	LEF-1 [T02905]	17	LXR-alpha:RXR-alpha [T0532	18	HNF-1A [T01211]	19	LyF-1 [T00479]	20	SRY [T00997]	21	TCF-4E [T02878	22	NFI/CTF [T	23	GR [T05076]
24	HNF-4alpha2 [T0	25	HNF-4alpha1 [T02429]	26	Crx [T03461]	27	AR [T00040]	28	XBP-1 [T00902]	29	c-Ets-1 68 [T00	30	NF-Y [T00	31	C/EBPgamma [T02028]
32	ER-alpha [T00261	33	c-Ets-2 [T00113]	34	HOXD8 [T01426]	35	HOXD8 [T01754]	36	GR-beta [T0192	37	POU1F1b [T01	38	POU1F1c [	39	HNF-3beta [T01049]
40	HNF-1A [T00368]	41	TFIID [T00820]	42	HNF-3alpha [T025	43	FOXP3 [T04280]	44	PR B [T00696]	45	PR A [T01661]	46	HNF-1C [T	47	HNF-1B [T01950]
48	HOXD9 [T01424]	49	HOXD10 [T01425]	50	HOXD9 [T01755]	51	HOXD9 [T01756]	52	HOXD10 [T0175	53	HOXD10 [T017	54	AP-2alpha	55	c-Jun [T00131]
56	IRF-1 [T00423]	57	NF-AT1 [T00550]	58	C/EBPalpha [T001	59	NF-1 [T00535]	60	HNF-4alpha1 [T	61	c-Jun [T00133]	62	AP-1 [T00	63	ENKTF-1 [T00255]
64	AhR [T01795]	65	AhR:Arnt [T05394]	66	Ik-1 [T02702]	67	HNF-4alpha [T038	68	USF-1 [T00875]	69	COUP-TF1 [T00	70	GR [T0033	71	PEA3 [T00685]
72	RXR-alpha [T013	73	RAR-beta [T00721]	74	PPAR-alpha:RXR-	75	VDR [T00885]	76	PXR-1:RXR-alph	77	GATA-3 [T0031	78	Elk-1 [T00	79	CAC-binding protein [T00076]
80	GATA-1 [T00305]	81	GATA-2 [T00308]	82	GATA-1 [T00306]	83	C/EBPbeta [T0045	84	E2F-1 [T01542]	85	POU2F2 (Oct-2	86	SREBP-1c	87	T3R-beta1 [T00851]
88	VDR [T01369]	89	IRF-2 [T00425]	90	IRF-2 [T01491]	91	STAT5A [T04683]	92	NF-kappaB1 [T	93	Egr-3 [T00243]	94	Egr-1 [T01	95	Egr-3 [T05889]
96	USF2 [T00878]	97	Pax-5 [T00070]	98	p53 [T00671]	99	NF-1/L [T00599]	100	Sp1 [T00759]	101	RAR-beta:RXR-	102	PU.1 [T00	103	ATF3 [T01313]
104	POU2F1 [T00641]	105	C/EBPbeta [T00017]	106	AP-1 [T00029]	107	CREB [T00163]	108	CREBbeta [T02	109	ATF-2 [T00167]	110	ATF [T000	111	RBP-Jkappa [T01616]
112	PU.1 [T02068]	113	MEF-2A [T01005]	114	MyoD [T00526]	115	RelA [T00594]	116	AP-2 [T00034]	117	E2F [T00221]	118	c-Ets-1 [T0	119	WT1 [T00899]
120	GCF [T00320]	121	EBF [T05427]	122	AP-2beta [T02469	123	ATF-1 [T00968]	124	CREB [T00164]	125	ETF [T00270]	126	Elk-1 [T05	127	HES-1 [T01649]
128	MAZ [T00490]	129	NF-kappaB [T00590]	130	IPF1 [T02057]	131	HNF-4alpha [T052	132	RAR-alpha1 [T0	133	CTF [T00174]	134	FOXF1 [T0	135	TBP [T00794]

Predicted by PROMO Nucleic Acids Res, 31, **13**, 3651-3653,2003

FigureS1 Predicted transcriptional Factors.

Figure S2

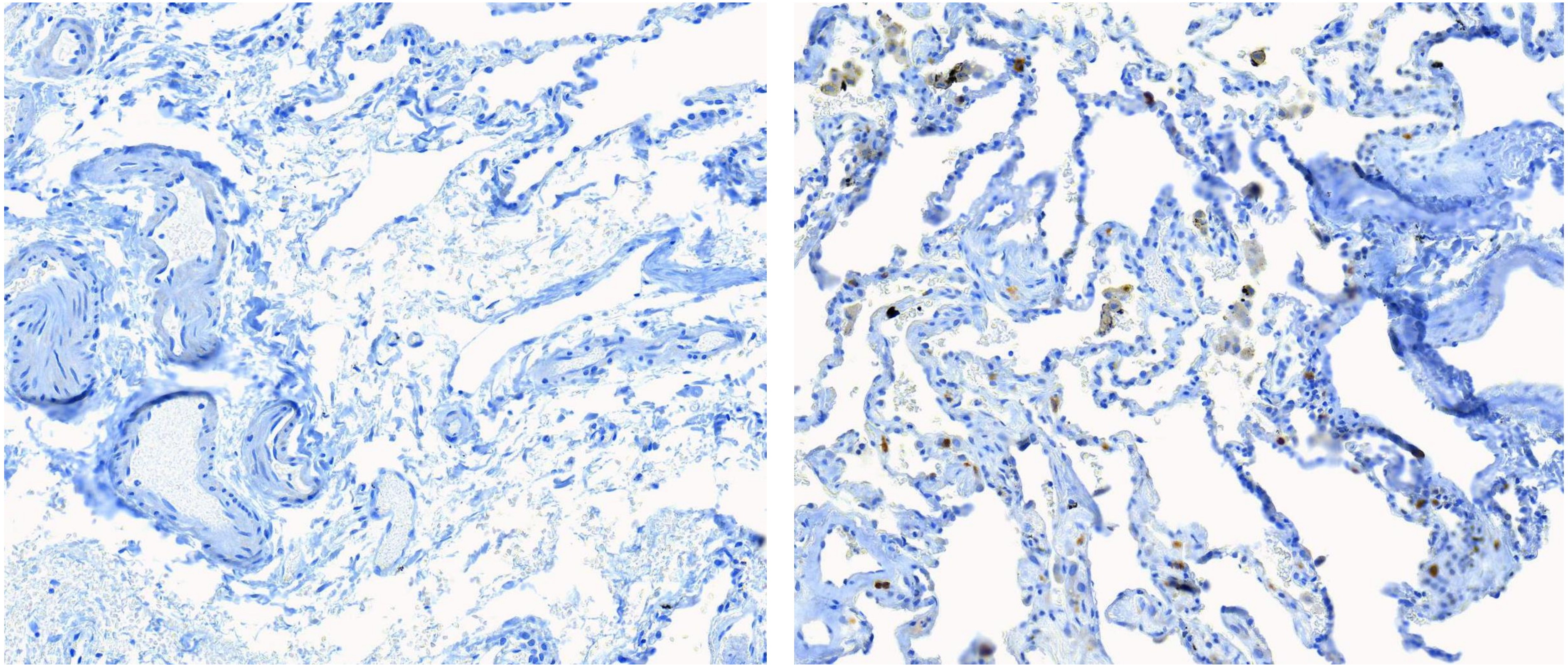

Figure S2 Representative IHC staining image for normal lung tissue. A,TFAP2A. B, TFAP2B.

Supplementary Table 1 Primers used in this study

Primers for qPCR		
Gene	F/R	sequence
USP22	F	CTGGAGCTGGACATGACCCCTT
	R	GTAGTGGCCACTCTCCAAGGTCC
TFAP2A	F	ACTCCTTACCTCACGCCATC
	R	TTGCTGTTGGACTTGGACAG
TFAP2B	F	CGTCCAGTCAGTTGATGC
	R	CCCAGGCCGTCTTTATT
PPAPA	F	CAGCTGCAAGATCCAGAAAA
	R	AGATCTTGGCATTCGTCCAA
cMyc	F	CCTGGTGCTCCATGAGGAGAC
	R	CAGACTCTGACCTTTTGCCAGG
NFYA	F	CCCAGCAGATCATCCA
	R	CAGTCTGTGCCACTTGCTGT
SP1	F	CAGGTGCACCCAATTCAA
	R	TCCACCTGCTGTGTCATCAT

Primers for clone USP22 promoter		
USP22PO	F1	CCGGGTACCCCTATTGGCCAGGTCCCTTTTTC
	F2	CCGGGTACCACCTTCAGTGATCCACCCACCTTG
	F3	CCGGGTACCTTCTTTCTTCCAGGGCTTCCAT
	F4	CCGGGTACCGAAGTTCAAAACCAGGCTGACG
	F5	CCGGGTACCCACGGAAAAGGAGGTGTGTGAT
	F6	CCGGGTACCTCTCAGTTTGGCTTGGGTCAGT
	F7	CCGGGTACC TGCAAACAGCTCCCGATTA
	R1	CCGCTCGAGACTCGCGCGCCCGTTAGGCTCT
	R0	CCGCTCGAGCGCGGAGGCCGGACAAAGATG
primers for clone overexpression plasmids		
FLAG-cMyc	F	CCGGGTACCACCATGGACTACAAGGATGACGATGACAAGGGACCCCTCAACGTTAGCTTCACCACA
	R	CCGCTCGAGCCTTACGCACAAGAGTTCCGTAGCTG
FLAG-TFAP2A	F	GGCAAGCTTCCACCATGGACTACAAAGACGATGACGACAAGTCCATACTTGCCAAAATGGG
	R	GCCGAATTCTCACTTTCTGTGCTTCTCCT
FLAG-TFAP2B	F	GCCGGATCCACCATGGACTACAAAGACGATGACGACAAGCACTCACCTCCTAGAGAC
	R	GCCGG GAATTC TCATTTCCTGTGTTTCTCCTCC

Supplementary Table 2 siRNAs used in this study

Gene Symbol	Full Gene Name	Gene ID	Sense siRNA Sequence	Antisense siRNA Sequence
AHR	aryl hydrocarbon receptor	196	GGCUCUUUCAAGAUAGUAAtt	UUACUAUCUUGAAAGAGCCct
JUN	jun proto-oncogene	3725	GGCACAGCUUAAACAGAAAtt	UUUCUGUUUAAGCUGUGCCac
TFAP2A	binding protein 2 alpha)	7020	AGAGAAAAACUGGACAAAAtt	UUUUGUCCAGUUUUUCUCUta
TFAP2B	protein 2 beta)	7021	AGUUCAACUUCGAAGUACAtt	UGUACUUCGAAGUUGAACUga
KLF12	Kruppel-like factor 12	11278	CAACUAUCCCGAUAUGGAAtt	UUCCAUAUCGGGAUAGUUGtg
ZEB1	zinc finger E-box binding homeobox 1	6935	GGAAGAACGUGACAGCACAtt	UGUGCUGUCACGUUCUUCCgc
ATF1	activating transcription factor 1	466	GAAUGUCGCAGAAAGAAGAtt	UCUUCUUUCUGCGACAUUCtc
ATF2	activating transcription factor 2	1386	GUGAUACUGUCAAAGGUCAtt	UGACCUUUGACAGUAUCACca
ATF3	activating transcription factor 3	467	GCAAAGUGCCGAAACAAGAtt	UCUUGUUUCGGCACUUUGCag
CEBPA	CCAAT/enhancer binding protein (C/EBP), alpha	1050	CCGCUCCAAUGCCUACUGAtt	UCAGUAGGCAUUGGAGCGGtg
CEBPB	CCAAT/enhancer binding protein (C/EBP), beta	1051	CCGCCUGCCUUUAAAUCCAtt	UGGAUUUAAAGGCAGGCGGcg
CEBPG	CCAAT/enhancer binding protein (C/EBP), gamma	1054	CGGAAUUAGUGUUAUCCAUtt	AUGGAUAACACUAAUUCCGtt
ETS1	(avian)	2113	GGACUCUUUUGAAAGCAUAtt	UAUGCUUUCAAAAGAGUCCtg
ETS2	(avian)	2114	GACAGAAGAUCAAUAUGAAtt	UUCAUAUUGAUCUUCUGUCtt
FOS	v-fos FBJ murine osteosarcoma viral oncogene homolog	2353	CACUCCAAGCGGAGACAGAtt	UCUGUCUCCGCUUGGAGUGta
MYB	v-myb avian myeloblastosis viral oncogene homolog	4602	GAAUUGCUCCUAAUGUCAAtt	UUGACAUUAGGAGCAAUUCta
MYC	v-myc myelocytomatosis viral oncogene homolog (avian)	4609	AGACCUUCAUCAAAAACAUtt	AUGUUUUUGAUGAAGGUCUcg
NR2F1	nuclear receptor subfamily 2, group F, member 1	7025	UCUCAUCCGCGAUAUGUUAtt	UAACAUAUCGCGGAUGAGAgt
CREB1	cAMP responsive element binding protein 1	1385	CAUUAGCCCAGGUAUCUAUtt	AUAGAUACCUGGGCUAAUGtg
E2F1	E2F transcription factor 1	1869	GUCACGCUAUGAGACCUCAtt	UGAGGUCUCAUAGCGUGACtt
EGR1	early growth response 1	1958	CAACGACAGCAGUCCCAUUtt	AAUGGGACUGCUGUCGUUGga
ELK1	ELK1, member of ETS oncogene family	2002	GGCCUUGCGGUACUACUAUtt	AUAGUAGUACCGCAAGGCCcg
ESR1	estrogen receptor 1	2099	ACAUCAUCUCGGUUCCGCAtt	UGCGGAACCGAGAUGAUGUag
TEAD2	TEA domain family member 2	8463	CCGUUCACCUUGUCACUGAtt	UCAGUGACAAGGUGAACGGtg
FOXA1	forkhead box A1	3169	GCAAUACUCUUAACCAUAAtt	UUAUGGUUAAGAGUAUUGCca
FOXA2	forkhead box A2	3170	GGACCUCAAGGCCUACGAAtt	UUCGUAGGCCUUGAGGUCCat
FOXA3	forkhead box A3	3171	ACUCCUACAUGACCCUGAAtt	UUCAGGGUCAUGUAGGAGUtg
FOXC1	forkhead box C1	2296	ACUCUCCAGUGAACGGGAAtt	UUCCCGUUCACUGGAGAGUtg
FOXC2	forkhead box C2 (MFH-1, mesenchyme forkhead 1)	2303	AGAAGGACGUGUCCAAGGAtt	UCCUUGGACACGUCCUUCUtt
FOXD1	forkhead box D1	2297	GAAAAAUGGUUAACAUGUUtt	AACAUGUUAACCAUUUUUCct
FOXD3	forkhead box D3	27022	GCCACUCUCCGGACAGUUUtt	AAACUGUCCGGAGAGUGGCac
FOXE1	forkhead box E1 (thyroid transcription factor 2)	2304	GCAAUUAUAUCAUAACUUAtt	UAAGUUAUGAUAUAAUUGCtg

FOXE3	forkhead box E3	2301	GGACACUUCUUGGAGAUUUtt	AAAUCUCCAAGAAGUGUCCtc
FOXF2	forkhead box F2	2295	GAAAAGAUUUCGUCCUCAAtt	UUGAGGACGAAAUCUUUUCtg
FOXG1	forkhead box G1	2290	GCAUCUACGAGUUCAUCAUtt	AUGAUGAACUCGUAGAUGCcg
FOXJ1	forkhead box J1	2302	CUAAUUAGAUGACAGCAAAtt	UUUGCUGUCAUCUAAUUAGca
FOXJ2	forkhead box J2	55810	GGAAGAAUUCAAUACGGCAtt	UGCCGUAUUGAAUUCUUCCaa
FOXJ3	forkhead box J3	22887	GGAUGGUAGUGAUAGCCCAtt	UGGGCUAUCACUACCAUCCtg
FOXK1	forkhead box K1	221937	GUCGCUCUAUCACAAAGAAtt	UUCUUUGUGAUAGAGCGACgt
FOXK2	forkhead box K2	3607	CACCAGCUACCAAUAAAAAtt	UUUUUAUUGGUAGCUGGUGtt
FOXL1	forkhead box L1	2300	ACAUGUUUGAGAACGGCAAtt	UUGCCGUUCUCAAACAUGUcc
FOXL2	forkhead box L2	668	CGAAGUUCCCGUUCUACGAtt	UCGUAGAACGGGAACUUCGcg
FOXM1	forkhead box M1	2305	GCUCAUACCUGGUACCUAUtt	AUAGGUACCAGGUAUGAGCtg
FOXN2	forkhead box N2	3344	GCAUUGAUCCAAAAGAAGAtt	UCUUCUUUUGGAUCAAUGCtg
FOXN3	forkhead box N3	1112	CAUUUUCCGUAUUUUGCAAtt	UUGCAAAAUACGGAAAAUGtt
FOXO1	forkhead box O1	2308	GGUGGAUGCUCAAUCCAGAtt	UCUGGAUUGAGCAUCCACCaa
FOXO3	forkhead box O3	2309	CUCACUUCGGACUCACUUAtt	UAAGUGAGUCCGAAGUGAGca
FOXO4	forkhead box O4	4303	CCGCGAUCAUAGACCUAGAtt	UCUAGGUCUAUGAUCGCGGca
FOXO6	forkhead box O6	100132074	ACGACUUCAUGGACAGCGAtt	UCGCUGUCCAUGAAGUCGUtg
FOXP1	forkhead box P1	27086	AGCUAACACUAAAUGAGAUtt	AUCUCAUUUAGUGUUAGCUgc
FOXP4	forkhead box P4	116113	GCCUAUUUCCGCAGAAACAtt	UGUUUCUGCGGAAAUAGGCga
FOXQ1	forkhead box Q1	94234	CACUAUAAAUGAUAUGACUtt	AGUCAUAUCAUUUAUAGUGtg
FOXR2	forkhead box R2	139628	GGAUGAAGAUAAUGCAAGAtt	UCUUGCAUUAUCUUCAUCCtt
C2orf3	chromosome 2 open reading frame 3	6936	GGAGUAUGAAAAAUACGUAtt	UACGUAUUUUUCAUACUCCct
NR3C1	(glucocorticoid receptor)	2908	GCAGGAUCAGAAGCCUAUUtt	AAUAGGCUUCUGAUCCUGCtg
HOXD9	homeobox D9	3235	GCUGUUCGCUGAAGGAGGAtt	UCCUCCUUCAGCGAACAGCct
IKZF1	IKAROS family zinc finger 1 (Ikaros)	10320	UGUGCUCAUGGUUCACAAAtt	UUUGUGAACCAUGAGCACAtt
IRF1	interferon regulatory factor 1	3659	CCUCUGAAGCUACAACAGAtt	UCUGUUGUAGCUUCAGAGGtg
IRF2	interferon regulatory factor 2	3660	GAGGAAUAUUGAAGGCAAAtt	UUUGCCUUCAAUAUUCCUCtt
NFE2L1	nuclear factor (erythroid-derived 2)-like 1	4779	GCUGCGAGAUGAGAACGGAtt	UCCGUUCUCAUCUCGCAGCcg
LEF1	lymphoid enhancer-binding factor 1	51176	GUUGCUGAGUGUACUCUAAtt	UUAGAGUACACUCAGCAACga
MAZ	transcription factor)	4150	CAAUCUCCGGAGGCACGAAtt	UUCGUGCCUCCGGAGAUUGta
MEF2A	myocyte enhancer factor 2A	4205	CCAACUCGGAUAUUGUUGAtt	UCAACAAUAUCCGAGUUGGtt
NFIC	nuclear factor I/C (CCAAT-binding transcription factor)	4782	AAAUUAGAGUGAACAAGAAtt	UUCUUGUUCACUCUAAUUUtc
NFIX	nuclear factor I/X (CCAAT-binding transcription factor)	4784	GAAUCCGGACAAUCAGAUAtt	UAUCUGAUUGUCCGGAUUCcg
NFKB1	B-cells 1	4790	GGCUCAUGUUUACAGCUUUtt	AAAGCUGUAAACAUGAGCCgc
NFYA	nuclear transcription factor Y, alpha	4800	CAAUACCACCGUAUUCUUAtt	UAAGAAUACGGUGGUAUUGtt
TP53	tumor protein p53	7157	GUAAUCUACUGGGACGGAAtt	UUCCGUCCCAGUAGAUUACca

PAX5	paired box 5	5079	GGCUCGUCGUACUCCAUCAtt	UGAUGGAGUACGACGAGCCgg
POU2F1	POU class 2 homeobox 1	5451	CGCAAAAUCUUCUAACGCAtt	UGCGUUAGAAGAUUUUGCGct
PPARA	peroxisome proliferator-activated receptor alpha	5465	GAUCAAGUGACAUUGCUAAtt	UUAGCAAUGUCACUUGAUCgt
RARA	retinoic acid receptor, alpha	5914	GAAGAUUACUGACCUGCGAtt	UCGCAGGUCAGUAAUCUUCat
RARB	retinoic acid receptor, beta	5915	GAACCGACAAAAGUAGAUAtt	UAUCUACUUUUGUCGGUUCct
RBPJ	kappa J region	3516	GGGAAGCUAUGCGAAAUUAtt	UAAUUUCGCAUAGCUUCCCta
RELA	(avian)	5970	CCCUUUACGUCAUCCCUGAtt	UCAGGGAUGACGUAAAGGGat
RXRA	retinoid X receptor, alpha	6256	UCGUCCUCUUUAACCCUGAtt	UCAGGGUUAAAGAGGACGAtg
SALL3	sal-like 3 (Drosophila)	27164	CGCGGUUUAUCGAGGAUAAtt	UUAUCCUCGAUAAACCGCGtg
SP1	Sp1 transcription factor	6667	GCAACAUGGGAAUUAUGAAtt	UUCAUAAUUCCCAUGUUGCtg
SRY	sex determining region Y	6736	CAACAGCGAUGAUUACAGUtt	ACUGUAAUCAUCGCUGUUGaa
STAT1	signal transducer and activator of transcription 1, 91kDa	6772	CCUACGAACAUGACCCUAUtt	AUAGGGUCAUGUUCGUAGGtg
TAF1B	polymerase I, B, 63kDa	9014	CAUUUGAUCCUAUAGCUAAtt	UUAGCUAUAGGAUCAAAUGtc
TCF3	binding factors E12/E47)	6929	CCUGCAGAGUAAGAUAGAAtt	UUCUAUCUUACUCUGCAGGcc
TCF4	transcription factor 4	6925	GAAGGAUAUCAAAUCAAUUtt	AAUUGAUUUGAUAUCCUUCtt
USF1	upstream transcription factor 1	7391	AGGUGGAAGAUCUUAAAAAtt	UUUUUAAGAUCUUCCACCUgt
USF2	upstream transcription factor 2, c-fos interacting	7392	CCCUUACUCUCCAAAAAUUtt	AAUUUUUGGAGAGUAAGGGtg
VDR	vitamin D (1,25- dihydroxyvitamin D3) receptor	7421	AGUUCAUUCUGACAGAUGAtt	UCAUCUGUCAGAAUGAACUcc
WT1	Wilms tumor 1	7490	GGACUGUGAACGAAGGUUUtt	AAACCUUCGUUCACAGUCCtt
XBP1	X-box binding protein 1	7494	CCUGCCUACUGGAUGCUUAtt	UAAGCAUCCAGUAGGCAGGaa
YY1	YY1 transcription factor	7528	AGCUUUUGUUGAGAGUUCAtt	UGAACUCUCAACAAAAGCUtt
